# In Vitro Inhibition of Phosphodiesterase 3B (PDE 3B) by Anthocyanin-Rich Fruit Juice Extracts and Selected Anthocyanins

**DOI:** 10.3390/ijms21186934

**Published:** 2020-09-21

**Authors:** Celina Göttel, Sonja Niesen, Vanessa Daub, Theresa Werle, Tamara Bakuradze, Peter Winterhalter, Elke Richling

**Affiliations:** 1 Division of Food Chemistry and Toxicology, Department of Chemistry, Technische Universität Kaiserslautern, Erwin-Schrödinger-Straße 52, D-67663 Kaiserslautern, Germany; goettel@chemie.uni-kl.de (C.G.); VanessaY@t-online.de (V.D.); werle@rhrk.uni-kl.de (T.W.); bakuradze@chemie.uni-kl.de (T.B.); 2 Institute of Food Chemistry, Technische Universität Braunschweig, Schleinitzstraße 20, D-38106 Braunschweig, Germany; s.niesen@tu-braunschweig.de (S.N.); p.winterhalter@tu-braunschweig.de (P.W.)

**Keywords:** phosphodiesterase, cyclic adenosine monophosphate, anthocyanins, red fruit juice, inhibition, HPLC-ESI-MS^n^, Folin-Ciocalteu

## Abstract

Phosphodiesterases (PDEs) are essential enzymes for the regulation of pathways mediated by cyclic adenosine monophosphate (cAMP). Secondary plant compounds like anthocyanins (ACs) can inhibit PDE activity and, consequently, may be beneficial for lipid metabolism. This study investigated 18 AC-rich juice extracts and pure reference compounds from red fruits for potential inhibitory effects on PDE 3B activity. Extracts were obtained through adsorption on Amberlite^®^ XAD 7 resin. Based on this screening, the chokeberry, blueberry, pomegranate, and cranberry extracts were active, with half maximal inhibitory concentrations (IC_50_) ranging from 163 ± 3 µg/mL to 180 ± 3 µg/mL. The ACs in these extracts, peonidin-3-glucoside and cyanidin-3-arabinoside, were the most active single compounds (IC_50_ = 56 ± 20 µg/mL, 108 ± 6 µg/mL). All extracts comprised high amounts of phenolic compounds, as determined by the Folin–Ciocalteu assay, ranging from 39.8 ± 1.5 to 73.5 ± 4.8 g gallic acid equivalents (GAE)/100 g extract. Pomegranate and chokeberry extracts exhibited the largest amounts of polyphenols (72.3 ± 0.7 g GAE/100 g, 70.6 ± 4.1 g GAE/100 g, respectively). Overall, our results showed that fruit juice extracts and their ACs can inhibit PDE activity. Any potential health benefits in vivo will be investigated in the future.

## 1. Introduction

The increased prevalence of obesity has become a major health problem worldwide. Abnormal or excessive fat accumulation is associated with higher risk of cardiovascular disease, type II diabetes, and other diseases, including some forms of cancer. In 2016, 39% of the world’s adult population was overweight and around 13% was obese [1].

Numerous polyphenols from fruits, including flavonoids like anthocyanins (ACs), have been studied extensively over recent decades because they mediate various biological effects [2]. ACs represent a class of secondary plant compounds that occur in many red fruits, such as cranberries, red grapes, black currants, and blueberries, and juices of the daily diet, where they contribute to their visual attractiveness. Total AC concentrations range considerably from 0.7 to 1480 mg/100 g of fresh weight in gooseberry and chokeberry, respectively [3]. The concentrations differ considerably, but the compositions of ACs in the fruits also vary. Six common aglycones (delphinidin, cyanidin, petunidin, pelargonidin, peonidin, and malvidin) were identified in all AC-rich plants, but with various sugar moieties and acylation patterns [3,4]. The different concentrations and compositions are thought to depend on both internal and environmental factors: genetic and agronomic factors, cultivation conditions (light intensity, irrigation, and temperature), as well as processing and storage conditions, respectively [5].

Besides the ACs, the abovementioned fruits also contain so-called copigments—colorless phenolic compounds such as phenolic acids, flavonols, and proanthocyanidins that can stabilize the colored structural form of the ACs and enhance their color intensity through intermolecular interactions or shifts in maximum absorption [4]. Several studies showed that ACs and copigments possess antioxidant, anti-inflammatory, and antidiabetic properties and are associated with numerous health benefits [6,7,8,9,10,11,12]. Reports also suggest that lipolytic activity can be influenced by polyphenols such as flavonoids and flavanones via inhibition of 3′,5′-cyclic adenosine monophosphate (cAMP)-phosphodiesterase, as demonstrated by an increasing cAMP level [13,14]. Cyclic AMP activates protein kinase A, leading to increased phosphorylation, which, consequently, stimulates activation of hormone-sensitive lipase as well as perilipin 1 and promotes lipolysis. During lipolysis, triglycerides located in fat cells (adipocytes) are degraded, releasing free fatty acids (FFAs) and glycerol [15,16]. These FFAs are bound to plasma albumin and transported to the liver, kidney, skeletal muscle, or myocardium, where they are used as oxidative fuel [17].

Phosphodiesterases (PDEs) are enzymes that catalyze the hydrolysis of phosphodiester bonds in the second messengers cAMP and 3′,5′-cyclic guanosine monophosphate (cGMP), resulting in the nucleoside 5′-monophosphates adenosine monophosphate and guanosine monophosphate, respectively. Together with adenylate and guanylate cyclases, PDEs play a major role in regulating signaling mediated by cAMP and cGMP. Compounds that can selectively inhibit PDEs are attractive for pharmaceutical research because PDE inhibition causes various beneficial physiological effects [18,19]. Certain constituents originating from foods can inhibit PDE. For example, caffeine was already recognized as a nonspecific inhibitor at the time of PDE discovery [20,21]. In addition, some classes of flavonoids (flavonols, flavones, ACs, flavanones, and flavanols) have shown PDE-inhibiting potential in several test systems [13,14,22,23,24,25]. The inhibitory effects of polyphenol-rich foods on PDE inhibition was demonstrated by inhibition of human cGMP-specific PDEs using red wine and grape skin extracts; this experiment was conducted using COS-7 cells that expressed the full-length cDNA of PDE 5A1 [26].

In the present study, we investigated the influence of 18 AC-rich fruit juice extracts obtained from pomegranates (*Punica granatum* L.; PG), blueberries (*Vaccinium myrtillus;* BB), cranberries (*Vaccinum macrocarpon*; CB), chokeberries (*Aronia melanocarpa*; CkB), black currants (*Ribes nigrum*; BC), elderberries (*Sambucus nigra*; EB), sour cherries (*Prunus cerasus*; SC), and red grapes (*Vitis vinifera*; RG) using a cell-free radiochemical cAMP PDE activity assay. The extracts were generated using Amberlite^®^ XAD 7 adsorber resin, and their AC and copigment profiles were analyzed using high-performance liquid chromatography with electrospray ionization mass spectrometry (HPLC-ESI-MS^n^) analyses. Furthermore, we investigated the effects of selected ACs on PDE activity to identify PDE-inhibiting compounds that may influence lipid metabolism in vivo. Additionally, the total amounts of phenolic compounds in the extracts were determined using the Folin–Ciocalteu assay [27].

## 2. Results

Eighteen fruit products, including pure fruit juices (not from concentrate (NFC)) and concentrates (juice concentrate (JC)), derived from pomegranates (PG1, PG2), blueberries (BB1, BB2), cranberries (CB1, CB2), chokeberries (CkB1, CkB2), black currants (BC1, BC2), elderberries (EB1, EB2), sour cherries (SC1, SC2, SC3), and red grapes (RG1, RG2, RG3) were subjected to extraction using Amberlite^®^ XAD 7 and the extracts were tested in vitro.

### 2.1. HPLC-ESI-MS^n^ Analysis

Table 1 and Table 2 show the major ACs and copigments identified in the extracts of the eight fruit varieties. A wide spectrum of AC-glycosides was identified in the extracts from the fruit juices, including glycosides of delphinidin, cyanidin, petunidin, peonidin, and malvidin. The selection of the major compounds is based on peak areas in the UV/Vis chromatogram. A complete list of all the anthocyanins detected is given in the Figures S1–S8, Tables S1, S3, S5, S7, S9, S11, S13 and S15.

The copigments identified included different types of phenolic compounds, such as flavonols (quercetin, myricetin, isorhamnetin), phenolic acids (chlorogenic acid, coumaric acid), and hydrolysable tannins (polymeric esters of gallic acid and sugars). A complete list of all the copigments detected is given in Figures S1–S8, Tables S2, S4, S6, S8, S10, S12, S14 and S16.

### 2.2. The Folin–Ciocalteu Assay

**Figure 1:**
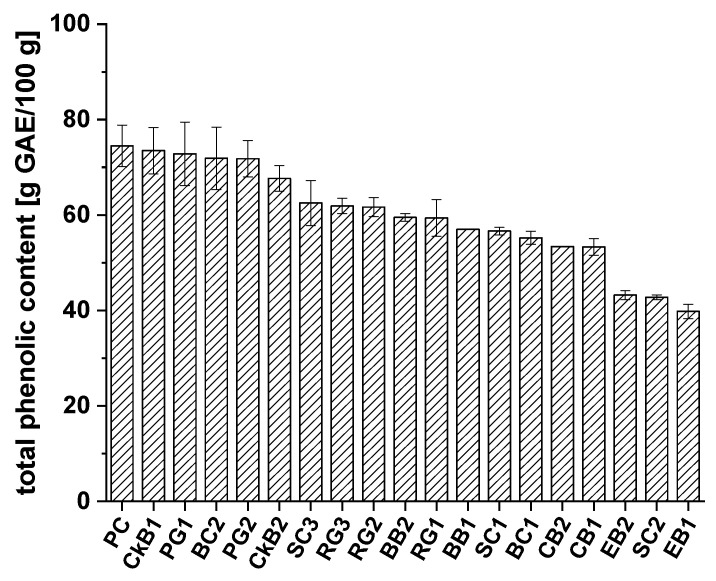
Total phenolic content of different extracts from chokeberry (CkB), cranberry (CB), pomegranate (PG), blueberry (BB), elderberry (EB), red grape (RG), sour cherry (SC), and black currant (BC) defined as gallic acid equivalents (g GAE/100g ± SD), compared to the positive control (PC), ascorbic acid.

The total phenolic content in fruit juice extracts from pomegranates (PG1, PG2), blueberries (BB1, BB2), cranberries (CB1, CB2), chokeberries (CkB1, CkB2), black currants (BC1, BC2), elderberries (EB1, EB2), sour cherries (SC1, SC2, SC3), and red grapes (RG1, RG2, RG3) was determined using the Folin–Ciocalteu assay. Gallic acid was used for calibration (gallic acid equivalents (GAE)) and ascorbic acid for the positive control (PC). The total phenolic content of each extract is presented in Figure 1.

**Figure 2:**
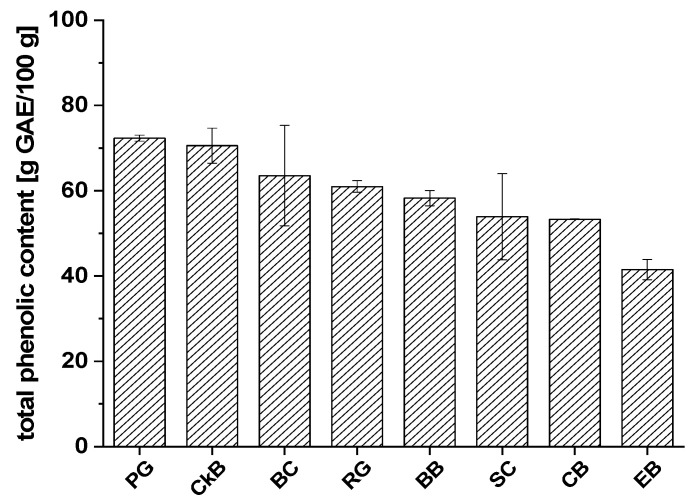
The average total phenolic contents of fruit juice extracts from chokeberry (CkB), cranberry (CB), pomegranate (PG), blueberry (BB), elderberry (EB), red grape (RG), sour cherry (SC), and black currant (BC) defined as gallic acid equivalents (g GAE/100 g ± SD).

All the fruit juice extracts examined showed high phenolic content, ranging from 39.8 to 73.5 g GAE/100 g of extract. The positive control, ascorbic acid (74.5 ± 4.3 g GAE/100 g), showed slightly higher phenolic content than the extracts from CkB1 (73.5 ± 4.9 g GAE/100 g) and PG1 (72.8 ± 6.7 g GAE/100 g). In comparison, the phenolic contents of extracts CkB2 and PG2 were 67.7 ± 2.7 g GAE/100 g and 71.8 ± 3.8 g GAE/100 g, respectively. BC2 extract also showed high total phenolic content (71.9 ± 6.5 g GAE/100 g). The following juice extracts exhibited phenolic contents in the medium range: SC3 (62.5 ± 4.7 g GAE/100 g), RG3 (61.9 ± 1.6 g GAE/100 g), RG2 (61.6 ± 2.0 g GAE/100 g), BB2 (59.5 ± 0.8 g GAE/100 g), RG1 (59.4 ± 3.8 g GAE/100 g), BB1 (57.0 ± 0.0 g GAE/100 g), SC1 (56.6 ± 0.8 g GAE/100 g), BC1 (55.2 ± 1.4 g GAE/100 g), CB2 (53.4 ± 0.0 g GAE/100 g), and CB1 (53.3 ± 1.7 g GAE/100 g). The lowest total phenolic content was found in EB2 (43.2 ± 0.9 g GAE/100 g), SC2 (42.8 ± 0.5 g GAE/100 g), and EB1 (39.8 ± 1.5 g GAE/100 g). For a better overview and comparison of the polyphenolic contents of the extracts, the average polyphenol contents were calculated from the data for each single fruit variety. The results are shown in Figure 2.

Extracts of PG (72.3 ± 0.7 g GAE/100 g), CkB (70.6 ± 4.1 g GAE/100 g), and BC (63.5 ± 11.7 g GAE/100 g) showed the highest total phenolic contents, followed by RG (61.0 ± 1.4 g GAE/100 g) and BB (58.4 ± 1.8 g GAE/100 g). The lowest total phenolic contents were found in SC (53.9 ± 10.1 g GAE/100 g), CB (53.3 ± 0.1 g GAE/100 g), and EB (41.5 ± 2.4 g GAE/100 g). Most of the fruit extract varieties had mean phenolic content values with small standard deviations; the BC and SC juice extracts were the exceptions. A possible explanation could be that there are significant differences between the types of BC and SC which we studied or that the fruits originated from cultivation regions with different environmental and/or climatic conditions.

### 2.3. Inhibitory Effects of Anthocyanin-Rich Fruit Juice Extracts on PDE 3B Activity In Vitro

The 18 extracts from red fruit juices or concentrates were tested for their ability to inhibit cAMP-specific PDE 3B activity in vitro. Data are presented as relative (rel.) PDE activity in percentage of the solvent control (2.5% dimethyl sulfoxide). Caffeine (1.21 mM), a well-known nonspecific PDE 3 inhibitor, was used as the positive control (PC) in this assay since it inhibited PDE activity by around 50% at a concentration of 1.21 mM.

**Figure 3:**
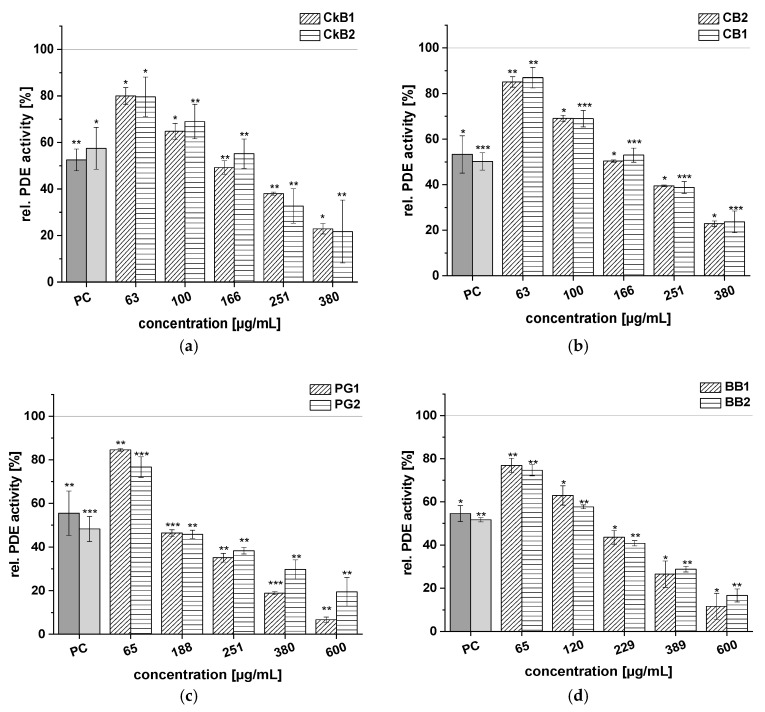
Results for tests of cAMP-specific phosphodiesterase (PDE) activity after incubation each with two chokeberry (CkB1, CkB2) (**a**), cranberry (CB1, CB2) (**b**), pomegranate (PG1, PG2) (**c**), and blueberry (BB1, BB2) (**d**) extracts. Positive control (PC) was caffeine (1.21 mM). Data are expressed as relative (rel.) PDE activity (in percentage of solvent control) as mean ± standard deviation of three or four independent experiments. The significance of differences between sample and solvent control (100%) was assessed using Student’s *t*-test. * *p* < 0.05; ** *p* < 0.01; *** *p* < 0.001.

The test results for the following extracts are presented in Figure 3: CkB1, CkB2, CB1, CB2, PG1, PG2, BB1, and BB2. Two different extracts from each fruit variety were tested to investigate potential differences between the PDE activity of extracts from the same fruit. All these fruit juice extracts showed significant, dose-dependent inhibition of the enzyme PDE 3B in vitro. For most of the fruit varieties, the PDE activities of the different juice extracts were in the same range.

Rel. PDE 3B activities of around 80% were shown by the CkB1 and CkB2 extracts (*c* = 63 µg/mL); the highest concentrations of these extracts (*c* = 380 µg/mL) decreased PDE activity to 22.9 ± 2.2% and 21.7 ± 13.5%, respectively, compared to the solvent control (Figure 3a). At the 166 µg/mL concentration, the two CkB extracts showed levels of activity comparable to the PC caffeine (around 50% PDE activity). A similar effect was observed for the two CB extracts (Figure 3b).

The PG extracts showed reduced activity of the PDE 3B enzyme when compared to the solvent control. At the highest concentration tested (*c* = 600 µg/mL), the PG1 extract showed a stronger inhibitory effect on PDE activity (6.7 ± 1.2%) than the PG2 extract (19.5 ± 6.6%; Figure 3c). Furthermore, incubation of PDE 3B with the two BB extracts led to diminished impact of both the BB1 (11.6 ± 6.1%) and BB2 (16.7 ± 3.0%) extracts on PDE activity at the highest concentration tested (*c* = 600 µg/mL), compared to the solvent control (Figure 3d). All the extracts of CkB, CB, PG, and BB demonstrated similar trends in PDE inhibition, ranging from around 20% to 80%.

**Figure 4:**
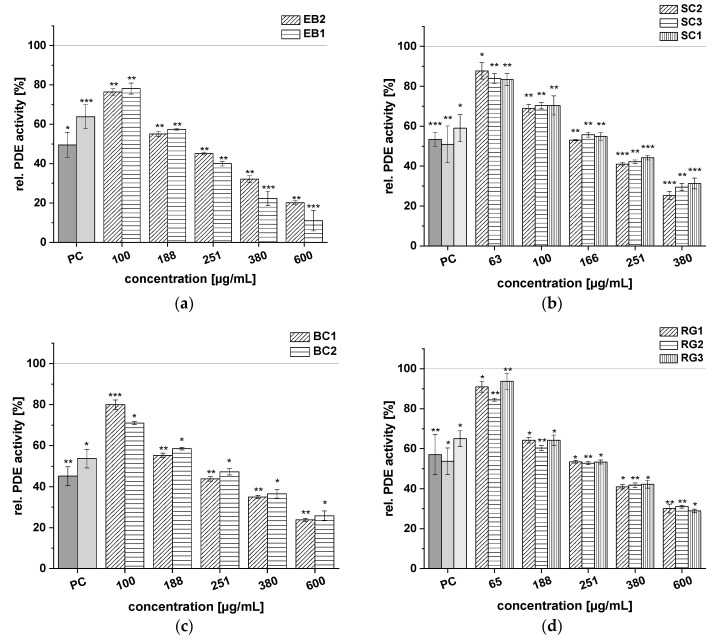
Results for tests of cAMP-specific phosphodiesterase (PDE) activity after incubation with elderberry (EB1, EB2) (**a**), sour cherry (SC1, SC2, SC3) (**b**), black currant (BC1, BC2) (**c**), and red grape (RG1, RG2, RG3) (**d)** extracts. Positive control (PC) was caffeine (1.21 mM). Data are expressed as relative (rel.) PDE activity (in percentage of solvent control) as mean ± standard deviation of three or four independent experiments. The significance of differences between sample and solvent control (100%) was assessed using Student’s *t*-test. * *p* < 0.05; ** *p* < 0.01; *** *p* < 0.001.

Test results for the following extracts are presented in Figure 4: EB1, EB2, SC1, SC2, SC3, BC1, BC2, RG1, RG2, and RG3. These fruit juice extracts also showed significant, dose-dependent inhibition of PDE activity, but the effects were less pronounced than the extracts from CkB, CB, PG, and BB shown in Figure 3. Extracts EB1 and EB2 both displayed concentration-dependent reduction of PDE enzyme activity (Figure 4a). At the highest concentration tested (*c* = 600 µg/mL), the EB1 extract was more potent than the EB2 extract, as demonstrated by a lower rel. PDE activity of 11.0 ± 5.2% for EB1 compared to 20.1 ± 1.0% for EB2. The three SC extracts inhibited PDE activity compared to the control, with activity levels ranging between 25.3 ± 2.0% and 31.3 ± 2.7% at a concentration of 380 µg/mL (Figure 4b). The two BC extracts reduced PDE activity significantly at all concentrations tested (*c* = 100–600 µg/mL), and, at a concentration of 251 µg/mL, the effect of both extracts on PDE activity was comparable to that of caffeine (around 50% PDE activity; Figure 4c). The three RG extracts (RG1, RG2, RG3) showed the weakest impact on PDE 3B activity of all the fruit extracts. Nevertheless, PDE activity after treatment with the RG extracts also diminished in a concentration-dependent manner, and minimum activity of around 30% was observed at the highest concentration tested (600 µg/mL; Figure 4d).

Based on the results of these PDE 3B activity assays, a half-maximal inhibitory concentration (IC_50_) was calculated for each of the 18 fruit juice extracts investigated. These IC_50_ values are presented in Table 3. The CkB extracts were the most active of the tested samples, exhibiting IC_50_ values between 163 ± 3 µg/mL and 167 ± 5 µg/mL. The BB and PG extracts demonstrated comparable inhibitory potential. The CB extracts revealed near-similar effects against PDE 3B (IC_50_ = 175 ± 1 µg/mL–180 ± 3 µg/mL). All extracts of CkB, BB, PG, and CB showed IC_50_ values less than 200 µg/mL, whereas greater IC_50_ values were determined for extracts of SC (SC1, SC2, SC3), followed by EB (EB1, EB2) and BC (BC1, BC2). IC_50_ values from the extracts of SC, EB, and BC ranged from 185 ± 1 µg/mL to 229 ± 3 µg/mL. The three RG extracts were the least active among all tested samples (IC_50_ = 276 ± 2 µg/mL–292 ± 3 µg/mL).

### 2.4. Inhibitory Effects of Selected Anthocyanins on PDE 3B Activity In Vitro

We tested the following eight commercially available ACs to obtain deeper insight into the inhibitory potential of extract constituents on PDE 3B: peonidin-3-glucoside, cyanidin-3-arabinoside, petunidin-3-glucoside, cyanidin-3-glucoside, cyanidin-3-galactoside, malvidin-3-glucoside, cyanidin-3-rutinoside, and cyanidin-3,5-diglucoside. We also tested the aglycon cyanidin to evaluate whether the effects on PDE 3B activity could be linked to the presence of ACs and to obtain structural information required for determining the efficacy of PDE 3B inhibition. Data are presented as rel. PDE activity in percent of the solvent control (dimethyl sulfoxide). Caffeine was used as the PC.

**Figure 5:**
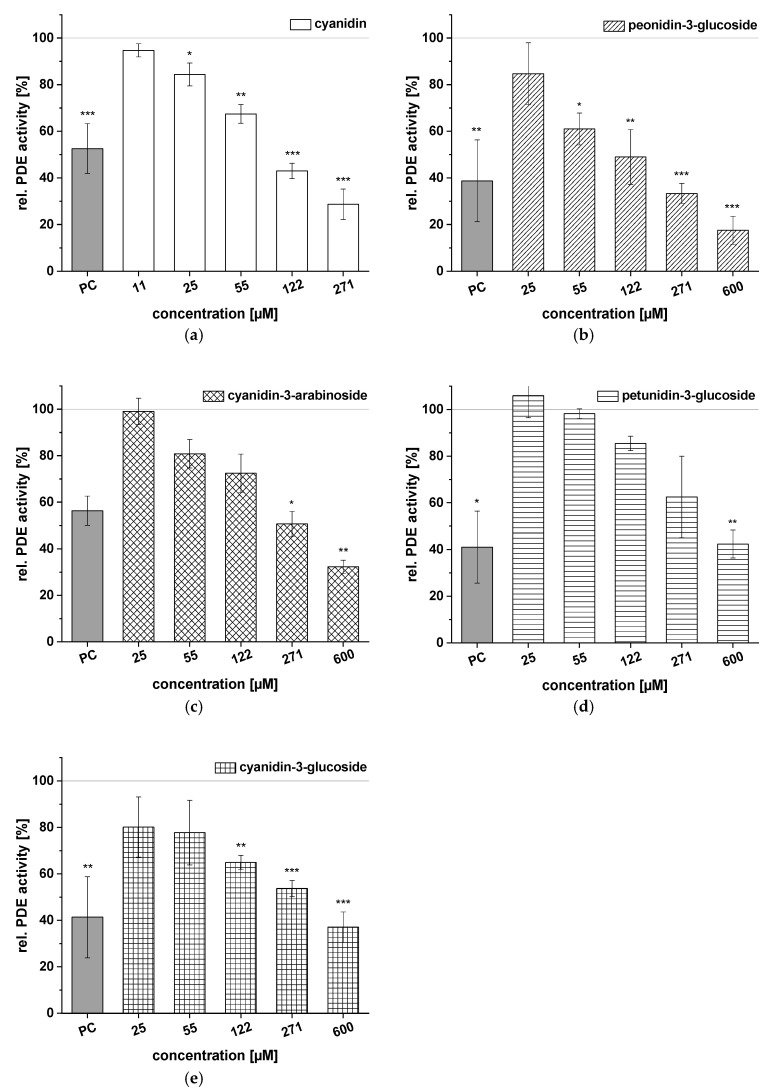
Results for tests of cAMP-specific phosphodiesterase (PDE) activity after incubation with cyanidin (**a**), peonidin-3-glucoside (**b**), cyanidin-3-arabinoside (**c**), petunidin-3-glucoside (**d**), and cyanidin-3-glucoside (**e**). Positive control (PC) was caffeine. Data are expressed as relative (rel.) PDE activity (in percentage of solvent control) as mean ± standard deviation of three or four independent experiments. The significance of differences between sample and solvent control (100%) was assessed using Student’s *t*-test. * *p* < 0.05; ** *p* < 0.01; *** *p* < 0.001.

The results of the evaluations of aglycon cyanidin and four ACs, peonidin-3-glucoside, cyanidin-3-arabinoside, petunidin-3-glucoside, and cyanidin-3-glucoside, on PDE activity are presented in Figure 5. All five compounds showed dose-dependent inhibition of the enzyme PDE 3B compared to the solvent control (100%). Cyanidin reduced PDE 3B activity in a dose-dependent manner from 94.7 ± 2.8% at the lowest tested concentration (*c* = 11 µM) to 28.7 ± 6.5% (*c* = 271 µM; Figure 5a). Peonidin-3-glucoside produced a similar effect, significantly diminishing the PDE activity at concentrations above 55 µM and showing impacts on PDE activity levels at a concentration of 271 µM that were comparable to those seen with caffeine (Figure 5b). Furthermore, when PDE was incubated with cyanidin-3-arabinoside, PDE activity was reduced to 32.3 ± 2.9% (*c* = 600 µM) of the solvent control (Figure 5c). The PDE activity (around 40%) of petunidin-3-glucoside and cyanidin-3-glucoside at the 600 µM concentration was in the same range (Figure 5d,e). Reduced PDE activities, compared to the solvent control, were already being detected with concentrations ranging from 25 to 271 µM of cyanidin-3-glucoside than of petunidin-3-glucoside. Generally, the aglycon cyanidin was found to be a more potent PDE inhibitor than its conjugated form.

**Figure 6:**
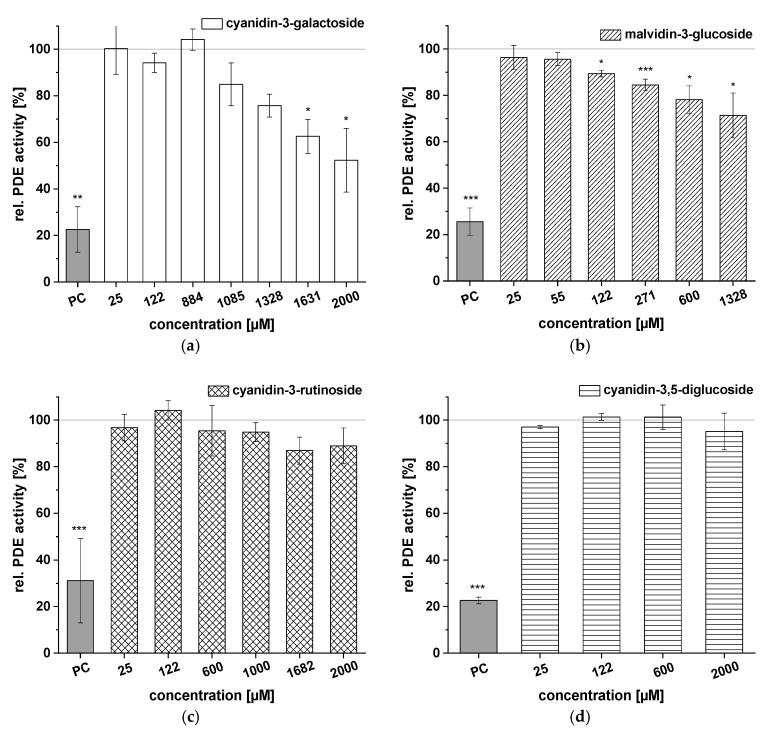
Results for tests of cAMP-specific phosphodiesterase (PDE) activity after incubation with cyanidin-3-galactoside (**a**), malvidin-3-glucoside (**b**), cyanidin-3-rutinoside (**c**), and cyanidin-3,5-diglucoside (**d**). Positive control (PC) was caffeine. Data are expressed as relative (rel.) PDE activity (in percentage of solvent control) as mean ± standard deviation of three or four independent experiments. The significance of differences between sample and solvent control (100%) was assessed using Student’s *t*-test. * *p* < 0.05; ** *p* < 0.01; *** *p* < 0.001.

Test results for the ACs cyanidin-3-galactoside, malvidin-3-glucoside, cyanidin-3-rutinoside, and cyanidin-3,5-diglucoside are illustrated in Figure 6. Cyanidin-3-galactoside had no influence on PDE activity at levels below 884 µM (Figure 6a), but effects were observed at higher concentrations (1085–2000 µM; 52.3 ± 13.7% PDE activity; *c* = 2000 µM). Malvidin-3-glucoside effects on PDE activity were concentration-dependent, with a minimum PDE activity of 71.4 ± 9.7% observed at a concentration of 1328 µM (Figure 6b). Cyanidin-3-rutinoside and cyanidin-3,5-diglucoside (*c* = 25–2000 µM) proved inactive, with no influence on PDE activity at any of the tested concentrations (Figure 6c,d).

We calculated the IC_50_ values of the ACs that we evaluated based on the results of the PDE activity assay summarized in Table 4. Cyanidin, the only aglycon tested, was the most potent inhibitor, with an IC_50_ value of 105 ± 11 µM, followed by peonidin-3-glucoside (IC_50_ = 120 ± 44 µM). Furthermore, when PDE was incubated with cyanidin-3-arabinoside and cyanidin-3-glucoside, IC_50_ values between 257 ± 13 µM and 299 ± 56 µM were calculated. Petunidin-3-glucoside showed a weaker effect on PDE inhibition, with an IC_50_ value of 448 ± 39 µM, and no IC_50_ values could be calculated for cyanidin-3-galactoside, cyanidin-3-rutinoside, cyanidin-3,5-diglucoside, and malvidin-3-glucoside.

## 3. Discussion

The aim of this work was to determine whether AC-rich fruit juice extracts and ACs present in the respective extracts could inhibit PDE 3B enzyme activity in vitro. Additionally, we analyzed the extracts using HPLC-ESI-MS^n^ and determined their total phenolic contents using Folin–Ciocalteu assay.

Total phenolic content of each extract was determined using the Folin–Ciocalteu assay, an established method for quantifying phenolics in different areas of food research [28]. All extracts of interest showed high total phenolic content, ranging from 39.8 to 73.5 g GAE/100 g of extract.

We must consider that the Folin–Ciocalteu assay measures all reducing compounds and not phenolic compounds specifically [28]. Moreover, gallic acid was used as a reference and calibration standard; therefore, the results for the extracts are not absolute values and should be regarded as approximate values for comparing the contents of extracts from the same fruit. We determined that the extract contents of the fruit juices that we investigated were 0.4 g/100 mL of the CB, RG, and SC juices, around 0.6 g/100 mL in the PG and BB juices, and up to 1.4 g/100 mL in the EB and CkB juices. Moreover, the total phenolic contents of the juices ranged between 0.2 and 0.5 g/100 mL, with the CkB juice extract showing up to 1 g/100 mL of total phenolic content. This last finding corresponds with studies by Esatbeyoglu et al. [29], who focused on the composition of the phenolic components in CkB juices.

All tested red fruit juice extracts exhibited dose-dependent PDE 3B inhibition. Of the extracts tested, CkB, PG, BB, and CB demonstrated the strongest PDE inhibition. In fact, at a concentration of 380 µg/mL, the CkB, PG, BB, and CB extracts inhibited PDE activity by around 80% (Figure 3). Based on the IC_50_ values of these extracts, the strength of the fruit extract inhibitory activity was ranked as follows: CkB > BB ≈ PG > CB > SC > EB > BC >> RG.

Not much research has been conducted on the influence of AC-rich fruit extracts on PDE inhibition. However, one report demonstrated that a polyphenolic citrus extract (a mixture of red orange, grapefruit, and orange) was a potent inhibitor (97% inhibition) of phosphodiesterase 3′,5′-cyclic nucleotide 5′nucleotidohydrolase [14]. Another study revealed that a Cabernet Sauvignon wine (containing 31.4 mg/L ACs) reduced human PDE 5A1 activity to 78.8 ± 2.0%, and grape skin extracts reduced PDE 5A1 activity to between 73.6 ± 5.9% and 85.7 ± 3.5% compared to the control, which had a concentration of 25 µg/mL (calculated as total phenols) [26].

In our study, we observed PDE 3B activity of 84.4 ± 0.8% due to the RG2 extract at a concentration of 65 µg/mL, which is comparable to the findings of the abovementioned study [26]. In addition, Röhrig and coworkers tested the following polyphenol-rich extracts on PDE isolated from the lung tumor xenograft cell line LXFL529L: artichoke (*Cynara scolymus*) extract, which had a significant inhibitory influence on PDE activity (IC_50_ = 0.9 ± 0.1 mg/mL); the flavones luteolin (IC_50_ = 41 ± 10 µM) and 3,4-dicaffeoylquinic acid (IC_50_ > 1.0 mM), and strawberry tree fruit (*Arbutus unedo*) extract, which showed no inhibitory effect on PDE at levels below 5 mg/mL. A plausible explanation for the lack of inhibition by the strawberry tree extract could be low AC content [25], as strawberry tree fruit has an AC content of only 0.51 mg per 100 g of fresh fruit [30].

We tested the following eight commercially available ACs to evaluate their effects on PDE 3B activity in connection with their presence in the extracts, as well as to obtain structural information required for the efficacy on PDE 3B: peonidin-3-glucoside, cyanidin-3-arabinoside, petunidin-3-glucoside, cyanidin-3-glucoside, cyanidin-3-galactoside, malvidin-3-glucoside, cyanidin-3-rutinoside, and cyanidin-3,5-diglucoside. In addition, the aglycon cyanidin was included in this test series.

Cyanidin was the most potent PDE 3B inhibitor in our study, with an IC_50_ value of 105 ± 11 µM. Marko and coworkers reported cyanidin-induced inhibition of cytosolic PDE activity in HT29 cells (IC_50_ ≈ 175 µM) [22]. Furthermore, our results showed that peonidin-3-glucoside, cyanidin-3-arabinoside, cyanidin-3-glucoside, and petunidin-3-glucoside showed potential for inhibiting PDE, with IC_50_ values ranging between 120 ± 44 µM and 448 ± 39 µM. No IC_50_ values could be determined for cyanidin-3-galactoside, malvidin-3-glucoside, cyanidin-3-rutinoside, and cyanidin-3,5-diglucoside due to their weak inhibitory effects. Cyanidin-3-glucoside inhibited PDE 3B by 63% at a concentration of 600 µM. Earlier publications reported that cyanidin-3-glucoside (diluted to 0.01% in dimethyl sulfoxide) resulted in 99% PDE inhibition [14].

We demonstrated that the aglycon cyanidin (IC_50_ = 105 ± 11 µM) and the cyanidin-monoglycosides (IC_50_ = 120 ± 44 µM–448 ± 39 µM) are more potent PDE inhibitors than cyanidin-disaccharides (no IC_50_ determinable). Previous investigations reported that the aglycon malvidin (IC_50_ = 24.9 µM) was the most potent inhibitor of PDE 5A1 and that glucosylation diminished its inhibitory effect (malvidin-3-glucoside IC_50_ = 35.4 µM) [26].

The ACs that we tested possessed different sugar moieties and aglycon substitution patterns. We investigated the influence of the aglycon on the levels of PDE inhibition using the following ACs: cyanidin-3-glucoside, malvidin-3-glucoside, peonidin-3-glucoside, and petunidin-3-glucoside. Based on their IC_50_ values, these ACs ranked as follows: peonidin-3-glucoside < cyanidin-3-glucoside < petunidin-3-glucoside < malvidin-3-glucoside. The number of hydroxy or methoxy groups on the B-ring obviously influences inhibitory potential. Peonidin-3-glucoside and cyanidin-3-glucoside (IC_50_ of 120 ± 44 µM and 299 ± 56 µM, respectively) each carry two substituents (hydroxy or methoxy groups) on the B-ring and have greater potential for PDE inhibition than the ACs with three B-ring substituents (petunidin-3-glucoside and malvidin-3-glucoside; IC_50_ = 448 ± 39 µM and not determined). The additional B-ring substituent leads to larger molecule size, which might cause steric hindrance, resulting in a poor fit of these ACs into the PDE 3B binding pocket.

Inconsistent with our findings, a previously published report showed that glucosides and aglycons both inhibited human PDE 5A1 activity in the following order of potency: malvidin > peonidin = delphinidin > petunidin > pelargonidin = cyanidin [26]. Comparable to these results, Marko and coworkers showed the structure-based impact of anthocyanidins on the inhibition of cytosolic PDE activity in HT29 cells, which could be summarized as follows: malvidin > peonidin > pelargonidin ≈ cyanidin > delphinidin. The authors concluded that the inhibitory potential of anthocyanidins on PDE increases with an increasing number of methoxy groups [22]. This observation could not be confirmed by our results. In fact, we found that malvidin-3-glucoside was the weakest inhibitor of PDE 3B.

The disparities among the studies could be due to the different test systems used (HT29 cells, human recombinant PDE 5A1 isoform prepared by expressing the full-length PDE 5A1 cDNA into COS-7 cells, or cell-free). In cells, the presence of various enzymes leads to metabolization of the ACs, which could explain the varying observations. In addition, Marko and coworkers only examined the aglycons, while, in this work, the respective anthocyanin-3-monoglucosides were also examined to determine their potential for PDE 3B inhibition. ACs and their respective aglycons might be unstable under certain conditions, and their degradation products might contribute to their inhibitory effects on PDE activity as well. Published literature reports the stability of selected ACs and cyanidin under cell culture conditions. Anthocyanidins proved to be highly unstable in cell culture medium compared to their respective glycosides. ACs are more stable than anthocyanidins, and the resulting degradation products may contribute to their inhibitory effects. For example, cyanidin degraded rapidly in cell culture medium and the corresponding phenolic acid, protocatechuic acid, was formed asynchronously [8].

In the study presented, we demonstrated that red fruit juice extracts and their ACs could affect PDE activity. Since the tested fruits also contain copigments that can stabilize the colored structural form of the ACs and enhance their color intensity through intermolecular interactions or shifts in maximum absorption [4], we analyzed the ACs and copigment profiles of each extract (Table 1 and Table 2) to correlate the effects of each extract with its phenolic composition.

The CkB extracts proved the most potent inhibitors (IC_50_ = 163 ± 3 µg/mL and 167 ± 5 µg/mL) and contained mostly cyanidin-3-galactoside and cyanidin-3-arabinoside. The potential of the CkB extracts for PDE 3B inhibition might be linked to cyanidin-3-arabinoside, which has an IC_50_ value of 108 ± 6 µg/mL. The PG extracts mainly contained cyanidin-3,5-diglucoside and cyanidin-3-glucoside. This study found no effect of cyanidin-3,5-diglucoside on PDE, but cyanidin-3-glucoside showed strong inhibitory potential (IC_50_ = 134 ± 25 µg/mL) and could possibly contribute to the inhibitory effects of the PG1 and PG2 extracts (IC_50_ = 169 ± 2 µg/mL and 174 ± 1 µg/mL, respectively). For the three RG extracts, IC_50_ values ranged from 276 ± 2 µg/mL to 292 ± 3 µg/mL, and malvidin-3-glucoside and peonidin-3-glucoside were the most abundant ACs. Our analyses imply that peonidin-3-glucoside (IC_50_ = 56 ± 20 µg/mL) might be responsible for the inhibition potential of the RG extracts since malvidin-3 glucose had only a small effect on PDE activity. The BB extracts contain mainly peonidin-3-galactoside and cyanidin-3-galactoside, among other ACs, and also inhibited PDE activity (IC_50_ = 174 ± 3 µg/mL and 165 ± 2 µg/mL). Since cyanidin-3-galactoside showed little PDE inhibition (Figure 6a), other compounds might be responsible for the inhibitory activity of the BB extracts.

In addition to ACs, the extracts contained numerous copigments (Table 2), which are different types of phenolic compounds, including flavonols (quercetin, myricetin, isorhamnetin), phenolic acids (chlorogenic acid, coumaric acid), and hydrolysable tannins (polymeric esters of gallic acid and sugars). Copigments could also influence PDE activity. Rauf et al. [31] examined the inhibitory activity of the phenolic acids chlorogenic acid and gallic acid against PDE 1 in vitro. The phenolic acids possessed IC_50_ values of 36.67 ± 1.60 µM and 325.19 ± 3.62 μM, respectively. Chlorogenic acid is a major copigment in the CkB and BB extracts that we studied and, thus, may contribute to their inhibitory activity against PDE 3B. In another study reported by Kuppusamy and Das [13], flavonoids were tested for their effects on phosphodiesterase from rat adipocytes. Quercetin, luteolin, and genistein showed inhibitory potencies comparable to or greater than 3-isobutyl-2-methylxanthine (IC_50_ = 30–50 µM), whereas myricetin or kaempferol had IC_50_ values of 125 µM and 58 µM, respectively [13]. We identified quercetin-derivatives in the CB, BB, EB, and SC extracts (Table 2), and these extracts had IC_50_ values ranging between 165 ± 2 µg/mL and 222 ± 3 µg/mL. Thus, the inhibitory potency of these extracts could be attributed to the presence of both copigments and ACs. Since we did not investigate the effects of copigments on PDE 3B activity, we cannot fully explain any reasons for the influence of the copigments at present and must address this matter in future studies.

Caffeine, the positive control for the PDE activity assay, is an unspecific PDE inhibitor. In our study, caffeine (*c* = 1.21 mM) inhibited PDE 3B by around 50%, confirming reported data demonstrating that commercially-available PDE was inhibited by 56% with caffeine (*c* = 0.01%, diluted in dimethyl sulfoxide) [14]. Montoya et al. showed dose-dependent PDE inhibition by caffeine concentrations ranging from 0.1 to 5 mM in human platelet lysates, in vitro, and determined an IC_50_ value of 0.7 mM for caffeine [32]. Furthermore, platelet PDE activity after coffee consumption by humans was tested, since caffeine is a major constituent of coffee, and the in vivo results revealed highly significant PDE inhibition after coffee intervention despite indirect dependence on the caffeine content of coffee.

Taken together, we characterized the red fruit juice extracts of interest using HPLC-ESI-MS^n^ analyses and identified different ACs and copigments. We also measured the total phenolic content of each extract using the Folin–Ciocalteu assay. Most importantly, we identified extracts from red fruits and single ACs with PDE inhibitory potential. The use of an in vitro model of PDE activity reveals only preliminary data, yet one must acknowledge that such in vitro results are the first insights into structure–activity relationships. These insights require further evaluation with additional future studies, preferably in vivo—for example, performing human intervention studies that measure PDE activity in isolated thrombocytes with and without these red fruit juice extracts.

## 4. Materials and Methods

### 4.1. Chemicals and Reagents

All chemicals and reagents used and purchased were of analytical quality. Adenosine monophosphate (AMP) was purchased from Alexis Biochemicals (Lörrach, Germany); barium hydroxide octahydrate (Ba(OH)_2_·8H_2_O), caffeine, cyclic adenosine monophosphate (cAMP), phosphodiesterase (PDE) 3B (recombinant, EC 3.1.4.17), gallic acid, and ascorbic acid from Sigma Aldrich (St. Louis, MO, USA); [2,8-^3^H]-3′5′-cyclic adenosine monophosphate ammonium salt, 9.25 MBq/mL, from Hartmann Analytic (Braunschweig, Germany). Tris-(hydroxymethyl)-aminomethane (TRIS), and hydrochloric acid were obtained from Carl Roth (Karlsruhe, Germany). The Folin–Ciocalteu reagent, magnesium chloride hexahydrate (MgCl_2_·6H_2_O), and zinc sulphate heptahydrate (ZnSO_4_·7H_2_O) were ordered from Merck (Darmstadt, Germany), and dimethyl sulfoxide (DMSO) was obtained from VWR Int. S.A.S. (Darmstadt, Germany). Peonidin-3-glucoside chloride, cyanidin-3-arabinoside chloride, petunidin-3-glucoside chloride, cyanidin-3-glucoside chloride, cyanidin-3-galactoside chloride, malvidin-3-glucoside chloride, cyanidin-3-rutinoside chloride, cyanidin-3,5-diglucoside chloride, and cyanidin chloride were supplied by PhytoLab (Vestenbergsgreuth, Germany).

### 4.2. Samples

All extracts were generated using Amberlite XAD 7 adsorber resin from Sigma Aldrich. The extracts were derived from either authentic pure fruit juices (NFC) or fruit juice concentrates (JC) from commercial sources: BB1 (blueberry JC), BB2 (blueberry NFC), BC1 (black currant NFC), BC2 (black currant JC), CB1 (cranberry JC), CB2 (cranberry JC), CkB1 (chokeberry JC), CkB2 (chokeberry JC), EB1 (elderberry JC), EB2 (elderberry JC), PG1 (pomegranate JC), PG2 (pomegranate JC), RG1 (red grape JC), RG2 (red grape NFC), RG3 (red grape JC), SC1 (sour cherry JC) and SC2 (sour cherry NFC), SC3 (sour cherry JC).

A column filled with the adsorber resin was washed with methanol and equilibrated with water. Juices were applied directly onto the column, and concentrates had to be diluted with 5 parts water prior to application. Next, the column was washed with water to flush out sugars, minerals, proteins, and salt, and then phenolic compounds were eluted with methanol/acetic acid (19/1; *v*/*v*). The solvents were evaporated in vacuo and the residue was freeze-dried. The so-obtained extracts were free of sugars and the pH was almost neutral.

### 4.3. HPLC-ESI-MS/MS Analysis

The HPLC system (1100/1200 series, Agilent, Waldbronn, Germany) includes a binary pump (G1312A), an autosampler (G1329B), and a DAD-detector (G1316A). It was coupled to an HCT Ultra Ion Trap mass spectrometer (Bruker Daltonics, Bremen, Germany) with an electrospray ionization source (ESI). The anthocyanins and copigments were separated on a Luna C18(2) 3 µ column (150 × 2.0 mm, Phenomenex (Torrance, CA, USA)) using water/acetonitrile/formic acid (95/3/2; *v*/*v*/*v*) (eluent A) and water/acetonitrile/formic acid (48/50/2; *v*/*v*/*v*) (eluent B) at a flow rate of 0.2 mL/min. Gradient elution was performed: 0 min 6% B, 30 min 35% B, 35 min 40% B, 45 min 90% B, 50 min 90% B, 55 min 30% B, 70 min 6% B. The ESI source was operated in alternating mode (+/−3000 V) (positive mode for anthocyanins and negative mode for copigments), using nitrogen as the nebulizer (50 psi) and drying gas (10 L/min, 365 °C). The sample extracts (around 2 mg) were dissolved in 2 mL of eluent A with a pH of 2.52 so that the flavylium cation form of the anthocyanins was stabilized. Aliquots of 5 µL of each sample were analyzed by the HPLC-PDA-ESI-MS/MS method described above according to Ostberg-Potthoff et al. [33] using the Bruker Hystar 3.2, Bruker ESICompass 1.3 for HCT/Esquire, and Data Analysis Version 3.0 software packages (Bruker Daltonics, Bremen, Germany).

### 4.4. The Folin–Ciocalteu Assay

The total phenolic content of each juice extract was determined using the Folin–Ciocalteu assay according to the method published by Singleton and Rossi [27]. The Folin–Ciocalteu reagent includes molybdatophosphoric acid and tungstophosphoric acid, which are reduced by the phenolic compounds and form a blue complex that can be photometrically quantified. Gallic acid was used for calibration in concentrations ranging from 10 to 60 mg/L. In each case, 200 μL of the sample solution (100 mg extract/1 L H_2_O), the gallic acid solutions, the positive control ascorbic acid (50 mg/L), and water for a blank were put into semi-micro cuvettes. One milliliter (1 mL) of Folin–Ciocalteu solution (from Merck KGaA, Darmstadt, Germany; diluted 1 to 10 with water) and, after no more than 8 min, 800 μL Na_2_CO_3_ solution (7.5 %) were added to each cuvette. Then, after exactly 2 h, the samples were measured at a wavelength of λ = 760 nm.

### 4.5. The cAMP-Specific PDE Activity Assay

The inhibitory effect of each extract on PDE 3B was measured according to the method published by Pöch et al. [34] with slight modifications [25,32]. One hundred microliters (100 µL) of the sample and 20 µL of PDE 3B (200 Units/mL) were incubated for 15 min at 4°C. Afterwards, a 50 µL volume of cAMP Mix (30 mM Tris/HCl pH 7.4, 9 mM MgCl_2_, 3 mM 5′AMP, 3 µM cAMP, 2.6 µCi/mL [2,8-^3^H]-cAMP) was added and the mixture incubated for 25 min at 37 °C to allow for reaction. The mixture was then put on ice to stop the reaction, and 250 µL of ZnSO_4_ (0.266 M) and Ba(OH)_2_ (0.266 M) were added. Then, the mixture was centrifuged at 13,000 *g* for 9 min at 25 °C, 450 µL of the supernatant was mixed with 3.5 mL of a scintillation cocktail, and the resulting radioactivity was measured using a liquid scintillation counter (Tri-Carb 2100 TR, Packard, Meriden, CT, USA or Tri-Carb 2810 TR, Perkin Elmer, Waltham, MA, USA). Sample stock solutions were prepared in DMSO and, afterwards, dissolved in assay buffer, which did not exceed a final concentration of 2.5% DMSO. The nonspecific PDE 3B inhibitor caffeine served as a positive control. Experiments were performed in triplicate, and IC_50_ values were determined after at least three independent experiments. The ranges of test concentrations of extracts and single substances were intentionally chosen to align with similar ranges used for previous studies (extracts < 600 µg/mL; ACs < 2000 µM) to facilitate comparison with previous experiments.

### 4.6. Statistical Analysis

The results of the cAMP-specific PDE activity assay were presented as the mean ± standard deviation of at least three independent experiments. Statistical analyses were conducted using the Analysis Tool in MS Excel 2016 (Microsoft, Redmond, WA) and Origin 2018G (OriginLab, Northampton, MA). Data were checked on normal distribution (David test) and homogeneity of variance (Fisher’s f-test). The data of samples treated with extracts were analyzed for significant differences (*p* < 0.05, *p* < 0.01, and *p* < 0.001) compared to the solvent treated control (DMSO) by one-sample Student’s *t*-test (one-sided).

## Figures and Tables

**Table 1:** Major anthocyanins in the extracts of chokeberry, cranberry, pomegranate, blueberry, elderberry, red grape, sour cherry, and black currant identified in this study using (a) HPLC-ESI-MS^n^ analysis and authentic references or (b) tentatively identified using HPLC-ESI-MS^n^ analysis and literature data. For details, see the Materials and Methods section, Section 4.3.

Red Fruit	Number of Anthocyanins Identified	Major Anthocyanins	[M + H]^+^ *m*/*z*	Fragments *m*/*z*
chokeberry	6	Cyanidin-3-galactoside ^(a)^	449	287
		Cyanidin-3-arabinoside ^(a)^	419	287
cranberry	7	Peonidin-3-galactoside ^(b)^	463	301
		Cyanidin-3-galactoside ^(b)^	449	287
pomegranate	4	Cyanidin-3,5-diglucoside ^(b)^	611	287
		Cyanidin-3-glucoside ^(a)^	449	287
blueberry	14	Peonidin-3-galactoside ^(b)^	463	301
		Cyanidin-3-galactoside ^(a)^	449	287
elderberry	4	Cyanidin-3-sambubioside ^(b)^	581	287
red grape	21	Malvidin-3-glucoside ^(a)^	493	331
		Peonidin-3-glucoside ^(a)^	463	301
sour cherry	4	Cyanidin-3-(2G-glucosylrutinoside) ^(b)^	757	287
		Cyanidin-3-rutinoside ^(b)^	595	287
black currant	5	Cyanidin-3-rutinoside ^(b)^	595	287
		Delphinidin-3-rutinoside ^(b)^	611	303

**Table 2:** Major copigments identified in the extracts of chokeberry, cranberry, pomegranate, blueberry, elderberry, red grape, sour cherry, and black currant identified in this study using (a) HPLC-ESI-MS^n^ analysis and authentic references or (b) tentatively identified using HPLC-ESI-MS^n^ analysis and literature data. For details, see the Materials and Methods section, Section 4.3.

Red Fruit	Number of Copigments Identified	Major Copigments	[M − H]^−^ *m*/*z*	Fragments *m*/*z*
chokeberry	10	Chlorogenic acid ^(a)^	353	191, 179, 161
		Neochlorogenic acid ^(a)^	353	191, 179, 135
cranberry	26	Quercetin-hexoside ^(b)^	463	301
		Myricetin-hexoside ^(b)^	479	316
pomegranate	28	Pedunculagine ^(b)^	783	301
		Punicalagin ^(a)^	1083	601
blueberry	17	Chlorogenic acid ^(a)^	353	191
		Cumaroyliridoid ^(b)^	535	371
		Quercetin-hexoside ^(b)^	463	301
elderberry	10	Quercetin-3-rutinoside ^(a)^	609	301
		Quercetin-3-glucoside ^(a)^	463	301
red grape	9	Isorhamnetin-hexoside ^(b)^	479	316
		Quercetin-hexoside ^(b)^	463	301
sour cherry	10	Quercetin-3-(2^G^-glucosylrutinoside) ^(b)^	771	301
		Quercetin-3-rutinoside ^(a)^	609	301
black currant	11	Isorhamnetin-rutinoside ^(b)^	625	317

**Table 3:** Half-maximal inhibitory concentrations (IC_50_) of the 18 different juice extracts tested using the PDE activity assay. Results are presented as mean ± SD (*n* = 3–4). JC (juice concentrate), NFC (not from concentrate).

Extract	Abbreviation	Product	IC_50_ (µg/mL)
chokeberry	CkB1	JC	163 ± 3
	CkB2	JC	167 ± 5
blueberry	BB1	JC	174 ± 3
	BB2	NFC	165 ± 2
pomegranate	PG1	JC	169 ± 2
	PG2	JC	174 ± 1
cranberry	CB1	JC	175 ± 1
	CB2	JC	180 ± 3
sour cherry	SC1	NFC	185 ± 1
	SC2	JC	195 ± 2
	SC3	JC	200 ± 2
elderberry	EB1	JC	222 ± 3
	EB2	JC	206 ± 2
black currant	BC1	NFC	212 ± 1
	BC2	JC	229 ± 3
red grape	RG1	JC	290 ± 2
	RG2	NFC	276 ± 2
	RG3	JC	292 ± 3

**Table 4:** Half-maximal inhibitory concentrations (IC_50_) of cyanidin and anthocyanins (ACs). Results are presented as means ± SD (*n* = 3–4). n.d. = not determined due to weak inhibitory effects.

Anthocyanin/Anthocyanidin	IC_50_ (µM)
cyanidin	105 ± 11
peonidin-3-glucoside	120 ± 44
cyanidin-3-arabinoside	257 ± 13
cyanidin-3-glucoside	299 ± 56
petunidin-3-glucoside	448 ± 39
cyanidin-3-galactoside	n.d.
cyanidin-3-rutinoside	n.d.
cyanidin-3,5-diglucoside	n.d.
malvidin-3-glucoside	n.d.

## Supplementary Materials

The following are available online at https://www.mdpi.com/1422-0067/21/18/6934/s1.

## Abbreviations

AC	anthocyanin
BB	blueberry
BC	black currant
cAMP	3′,5′-cyclic adenosine monophosphate
CB	cranberry
cGMP	3′,5′-cyclic guanosine monophosphate
CkB	chokeberry
DMSO	dimethyl sulfoxide
EB	elderberry
FFA	free fatty acid
GAE	gallic acid equivalents
HPLC-ESI-MS^n^	high-performance liquid chromatography with electrospray ionization mass spectrometry
IC_50_ value	half maximal inhibitory concentration
JC	juice concentrate
n.d.	not determined
NFC	not from concentrate, direct juices
PC	positive control
PDE	phosphodiesterase
PG	pomegranate
rel.	relative
RG	red grape
SC	sour cherry
