# Supplementary material for: In Vitro Inhibition of Phosphodiesterase 3B (PDE 3B) by Anthocyanin-Rich Fruit Juice Extracts and Selected Anthocyanins
Source: Int J Mol Sci. 2020 Sep 21;21(18):6934. doi: 10.3390/ijms21186934 (PMC7555035; doi:10.3390/ijms21186934)
Supplement: Supplementary file 1 [file ijms-21-06934-s001.pdf]

International Journal of
Molecular Sciences

MDPI

HPLC-DAD chromatograms and isocontour plots for each juice type and complete list of the anthocyanins and copigments detected

Chokeberry

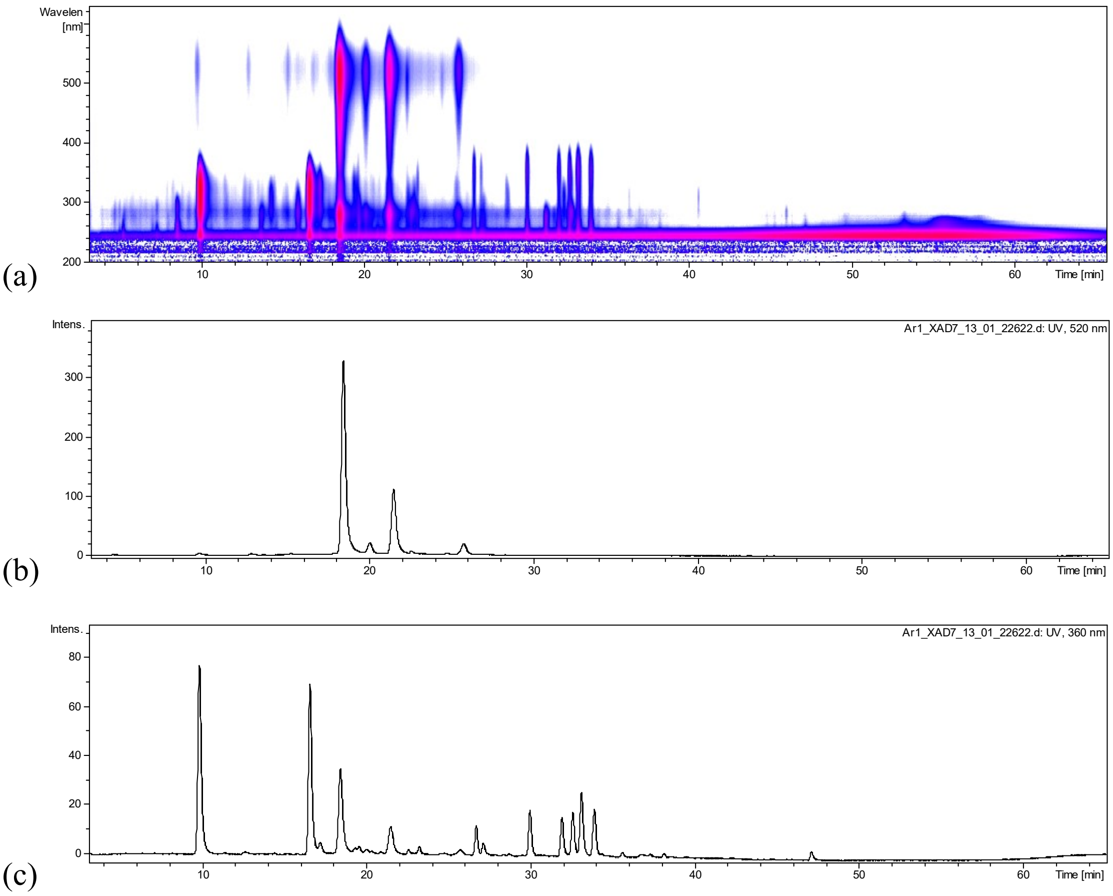

Figure S1. Isocontour plot from λ 200 to 600 (a) and DAD chromatograms at a wavelength of λ 520 (b) and λ 360 (c) of the high-performance liquid chromatography electrospray ionization mass spectrometry (HPLC-ESI-MS/MS) chromatograms of the XAD 7 extract of CkB1. Peak identification is given in Tables 1 and 2.

Table S1. HPLC-ESI-MS/MS data of the XAD 7 extract of CkB1, identified anthocyanins and their absorption maxima λ_{max}.

Peak No.	Retention time (min)	$[M-H]^-$ *m/z*	Fragments *m/z*	λ_{max}	Anthocyanin	Identification
1	9,9	737	575, 287	522	Cyanidin-derivative	C
2	12,9	707	575, 287	523	Cyanidin-derivative	C
3	18,4	449	287	515	Cyandin-3-galactoside	A
4	20,0	449	287	515	Cyanidin-3-glucoside	A
5	21,5	419	287	515	Cyanidin-3-arabinoside	A
6	25,8	419	287	517	Cyanidin-3-xyloside	B

Compounds were identified by (A) mass spectral data and comparison with authentic reference, (B) mass spectral data and literature data or (C) mass data only.

Table S2. HPLC-ESI-MS/MS data of the XAD 7 extract of CkB1, identified copigments and their absorption maxima λ_{max}.

Peak No.	Retention time (min)	$[M-H]^-$ *m/z*	Fragments *m/z*	λ_{max}	Copigment	Identification
1	10,0	353	191,179,135	323	Neochlorogenic acid	A
2	16,6	353	191, 179, 161	324	Chlorogenic acid	A
3	17,2	353	191	324	Cryptochlorogenic acid	A
4	26,8	625	301	351	Quercetin-dihexoside	C
5	27,2	625	301	349	Quercetin-dihexoside	C
6	29,9	595	301	352	Quercetin-3-vicianoside	B
7	32,0	609	301	351	Quercetin-3-robinobioside	B
8	32,6	609	301	349	Quercetin-3-rutinoside	A
9	33,2	463	301	352	Quercetin-3-galactoside	B
10	33,9	463	301	352	Quercetin-3-glucoside	A

Compounds were identified by (A) mass spectral data and comparison with authentic reference, (B) mass spectral data and literature data or (C) mass data only.

Cranberry

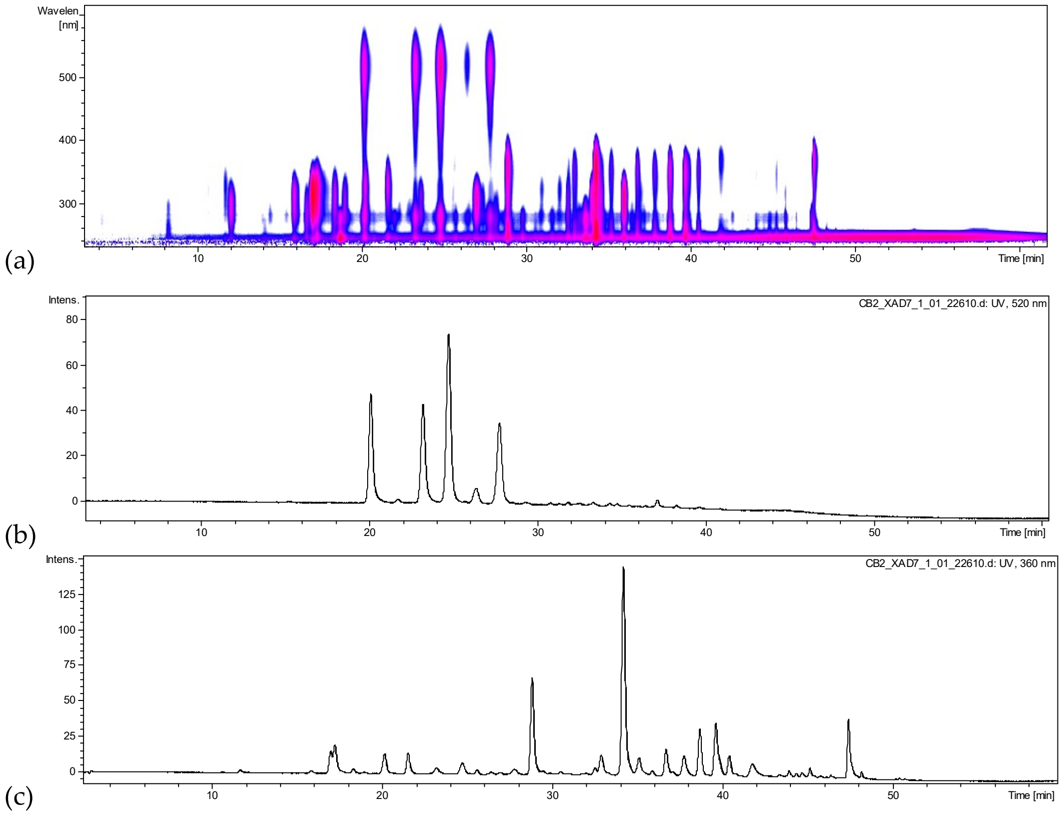

Figure S2. Isocontour plot from λ 200 to 600 (a) and DAD chromatograms at a wavelength of λ 520 (b) and λ 360 (c) of the high-performance liquid chromatography electrospray ionization mass spectrometry (HPLC-ESI-MS/MS) chromatograms of the XAD 7 extract of CB2. Peak identification is given in Tables 3 and 4.

Table S3. HPLC-ESI-MS/MS data of the XAD 7 extract of CB2, identified anthocyanins and their absorption maxima λ_{max}.

Peak No.	Retention time (min)	$[M+H]^+$ *m/z*	Fragments *m/z*	λ_{max}	Anthocyanin	Identification
1	20,2	449	287	515	Cyanidin-3-galactoside	A
2	21,9	449	287	524	Cyanidin-3-glucoside	A
3	23,3	419	287	516	Cyanidin-3-arabinoside	A
4	24,8	463	301	516	Peonidin-3-galactoside	B
5	26,5	463	301	520	Peonidin-3-glucoside	A
6	27,8	433	301	516	Peonidin-3-arabinoside	B
7	29,5	463	331	528	Malvidin-3-arabinoside	B

Compounds were identified by (A) mass spectral data and comparison with authentic reference, (B) mass spectral data and literature data or (C) mass data only.

Table S4. HPLC-ESI-MS/MS data of the XAD 7 extract of CB2, identified copigments and their absorption maxima λ_{max}.

Peak No.	Retention time (min)	$[M-H]^-$ *m/z*	Fragments *m/z*	λ_{max}	Copigment	Identification
1	15,9	341	179, 135	313	Caffeic acid hexoside	B
2	17,0	325	163	315	Coumaric acid hexosid	B
3	17,3	325	145	322	Coumaric acid hexosid	B
4	17,5	353	191	322	Chlorogenic acid	A
5	18,6	355	193	328	Ferulic acid	A
6	19,2	577	407	308	Proanthocyanidin dimer	B
7	19,8	385	223	294	Sinapic acid hexosde	B
8	22,5	335	179	325	caffeoylshikimic acid	B
9	23,0	337	191	312	Coumaroylquinic acid	B
10	24,0	863	711	310	Proanthocyanidin trimer	B
11	28,9	479	316	354	Myricetin-hexoside	B
12	29,3	449	316	354	Myricetin-xyloside	B
13	30,8	493	330	357	Laricitrin-hexoside	B
14	32,0	535	371	351	Coumaroyl Iridoid hexoside	B
15	32,8	537	373	311	Coumaroyl-dihydromonotropein	B
16	34,2	463	301	352	Quercetin-hexoside	B
17	35,1	463	301	355	Quercetin-hexoside	B
18	36,7	433	301	351	Quercetin-pentoside	B
19	37,8	433	301	350	Quercetin-pentoside	B
20	38,7	433	301	351	Quercetin-pentoside	B
21	39,6	447	301	346	Quercetin-rhamnoside	B
22	40,4	507	344	352	Syringetin-hexoside	B
23	41,8	317	179	368	Myricetin	A

24	42,3	447	314	355	Isorhamnetin-pentoside	B
25	43,0	477	344	351	Syringetin-pentoside	B
26	47,4	301	179	368	Quercetin	A

Compounds were identified by (A) mass spectral data and comparison with authentic reference, (B) mass spectral data and literature data or (C) mass data only.

Pomegranate

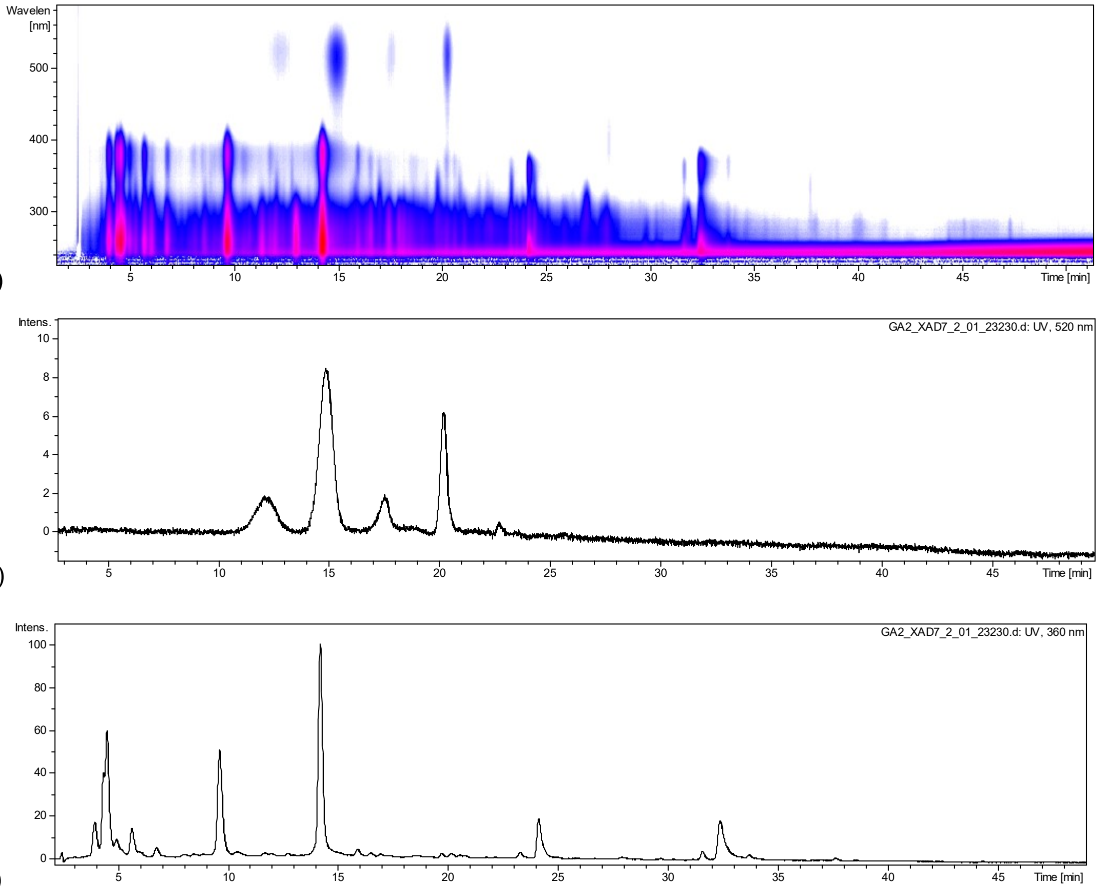

(a) (b) (c)

Figure S3. Isocontour plot from λ 200 to 600 (a) and DAD chromatograms at a wavelength of λ 520 (b) and λ 360 (c) of the high-performance liquid chromatography electrospray ionization mass spectrometry (HPLC-ESI-MS/MS) chromatograms of the XAD 7 extract of PG2. Peak identification is given in Tables 5 and 6.

Table S5. HPLC-ESI-MS/MS data of the XAD 7 extract of PG2, identified anthocyanins and their absorption maxima λ_{max}.

Peak No.	Retention time (min)	$[M+H]^+$ *m/z*	Fragments *m/z*	λ_{max}	Anthocyanin	Identification
1	12,1	627	303	523	Delphinidin-3,5-diglucoside	B
2	14,9	611	287	514	Cyanidin-3,5-diglucoside	A
3	17,4	595	271	514	Delphinidin-3-glucoside	A
4	20,3	449	287	517	Cyanidin-3-glucoside	A

Compounds were identified by (A) mass spectral data and comparison with authentic reference, (B) mass spectral data and literature data or (C) mass data only.

Table S6. HPLC-ESI-MS/MS data of the XAD 7 extract of PG2, identified copigments and their absorption maxima λ_{max}.

Peak No.	Retention time (min)	[M-H]⁻ *m/z*	Fragments *m/z*	λ_{max}	Copigment	Identification
1	3,4	783	721,601	377	Pedunculagin I	B
2	3,7	1101	781, 601	377	Punicalin-derivative	C
3	308	649	605, 301		Trisgalloyl-glucoside	B
4	3,9	781	601,271	378	Punicalin I	A
5	4,4	781	601,299	377	Punicalin II	A
6	4,8	1083	601		Punicalagin I	A
7	5,4	783	299,601	376	Pedunculagin II	B
8	7,9	783	301	377	Pedunculagin III	B
9	9,5	933	451	372	Galloyl-O-punicalin	B
10	10,9	469	425	371	Valonic acid bilactone	B
11	11,4	951	907	373	Granatin B	B
12	12,5	951	783	377	HHDP-valoneoyl-glucoside	B
13	13,3	1083	601	378	Punicalagin II	A
14	15,4	799	301	376	Ellagic acid derivative	C
15	15,8	1085	451	375	Digalloyl-gallagyl-hexoside	B
16	16,2	799	301	375	Granatin A	B
17	17,6	325	145	312	Coumaric acid hexoside	B
18	18,8	801	347	365	Digalloyl-HHDP-glucuronide	B
19	20,0	449	287	322	Dihydrokaempferol-hexoside	B
20	20,1	355	193	327	Ferulic acid hexoside	B
21	21,4	633	301	370	Galloyl-HHDP-glucoside	B
22	23,0	635	465	322	Tri-O-galloyl-glucoside	B
23	24,9	463	301	360	Ellagic acid hexoside	B
24	26,1	953	301	332	Galloyl-bis-HDDP-glucoside	B
25	29,5	447	301	360	Quercetin-3-rhamnoside	B
26	30,1	787	635	320	Tetra-O-galloyll-glucoside	B
27	32,3	491	328	369	Dimethyl ellagic acid hexoside	B
28	33,0	301	229	366	Ellagic acid	A

Compounds were identified by (A) mass spectral data and comparison with authentic reference, (B) mass spectral data and literature data or (C) mass data only.

Blueberry

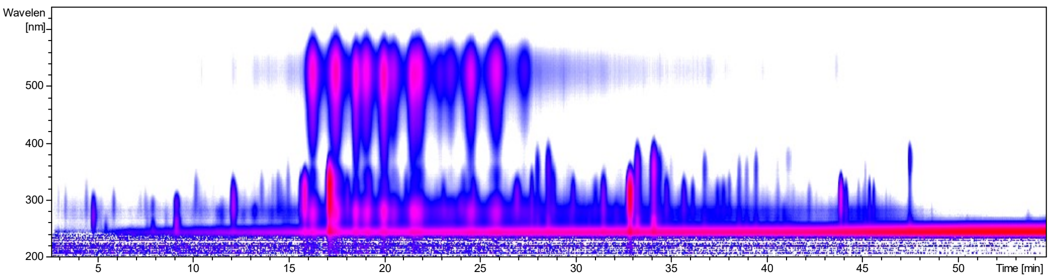

(a)

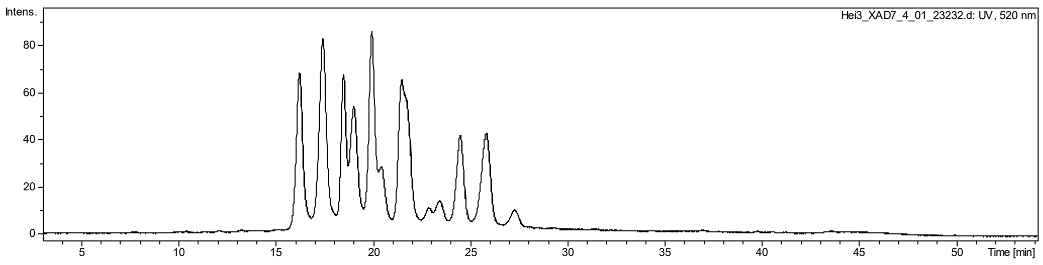

(b)

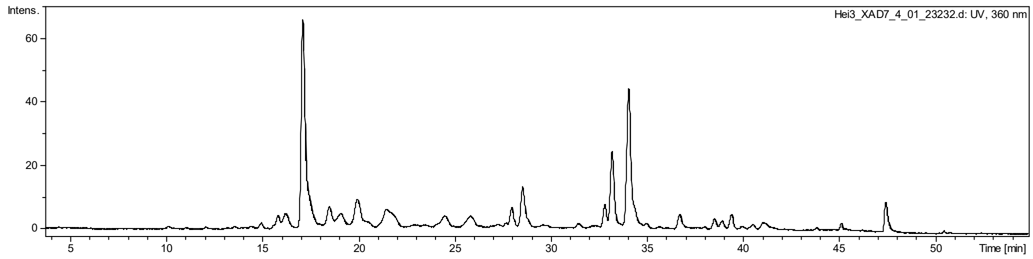

(c)

Figure S4. Isocontour plot from λ 200 to 600 (a) and DAD chromatograms at a wavelength of λ 520 (b) and λ 360 (c) of the high-performance liquid chromatography electrospray ionization mass spectrometry (HPLC-ESI-MS/MS) chromatograms of the XAD 7 extract of BB2. Peak identification is given in Tables 7 and 8.

Table S7. HPLC-ESI-MS/MS data of the XAD 7 extract of BB2, identified anthocyanins and their absorption maxima λ_{max}.

Peak No.	Retention time (min)	$[M+H]^+$ m/z	Fragments m/z	λ_{max}	Anthocyanin	Identification
1	16,3	465	303	522	Delphinidin-3-galactoside	B
2	17,4	465	303	522	Delphinidin-3-glucoside	A
3	18,6	449	287	515	Cyanidin-3-galactoside	A
4	19,1	435	303	523	Delphinidin-3-arabinoside	B
5	20,0	449	287	515	Cyanidin-3-glucoside	A
6	20,6	479	317	522	Petunidin-3-galactoside	B
7	21,4	419	287	517	Cyanidin-3-arabinoside	A
8	21,9	479	317	523	Petunidin-3-glucoside	A
9	22,9	463	301	519	Peonidin-3-galactoside	B
10	23,5	449	317	524	Petunidin-3-arabinoside	B
11	24,4	493	331	519	Malvidin-3-galactoside	B
12	24,5	463	301	518	Peonidin-3-glucoside	A
13	25,8	493	331	524	Malvidin-3-glucoside	A
14	27,2	463	331	526	Malvidin-3-arabinoside	B

Compounds were identified by (A) mass spectral data and comparison with authentic reference, (B) mass spectral data and literature data or (C) mass data only.

Table S8. HPLC-ESI-MS/MS data of the XAD 7 extract of BB2, identified copigments and their absorption maxima λ_{max}.

Peak No.	Retention time (min)	[M-H]- *m/z*	Fragments *m/z*	λ_{max}	Copigment	Identification
1	15,9	341	179	312	Caffeic acid hexoside	B
2	17,2	353	191	324	Chlorogenic acid	A
3	19,2	447	284	322	Kaempferol-hexoside	B
4	28,1	479	316	343	Myricetin-hexoside	B
5	28,5	479	316	348	Myricetin-hexoside	B
6	31,5	535	371	311	Coumaroyl Iridoid hexoside	B
7	32,4	463	301	348	Quercetin-hexoside	B
8	32,7	609	301	311	Quercetin-rutinoside	A
9	33,1	535	371	311	Coumaroyl Iridoid hexoside	B
10	34,0	463	301	352	Quercetin-hexoside	B
11	34,2	477	301	352	Quercetin-glucuronide	B
12	36,9	433	300,301	351	Quercetin-pentoside	B
13	38,1	411	145	-	Cuomaric acid derivative	B
14	38,7	447	300,301	343	Quercetin-desoxyhexoside	B
15	39,5	507	344	343	Syringetin-hexoside	B
16	45,2	591	447	306	Quercetin-3-(4-HMG)-rhamnoside	B
17	47,5	301	151	369	Quercetin	A

Compounds were identified by (A) mass spectral data and comparison with authentic reference, (B) mass spectral data and literature data or (C) mass data only. HMG = 3-hydroxy-3-methylglutaroyl.

Elderberry

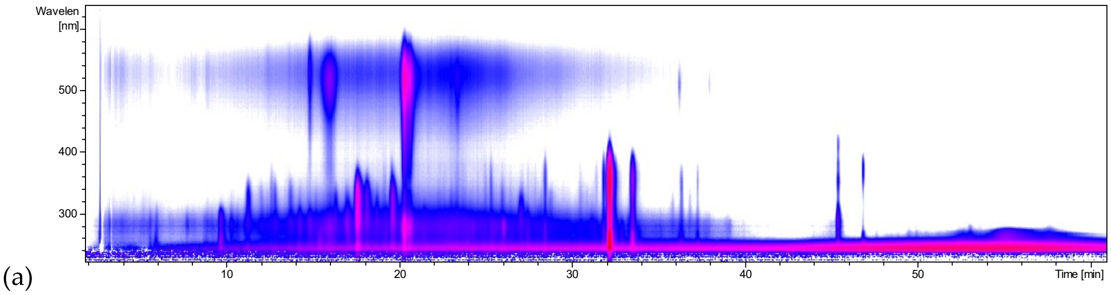

(a)

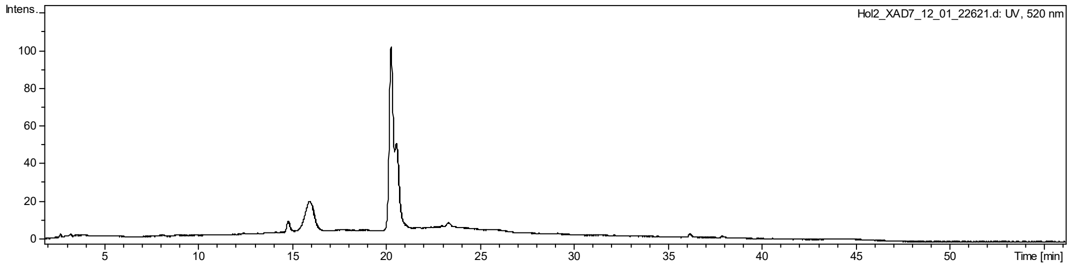

(b)

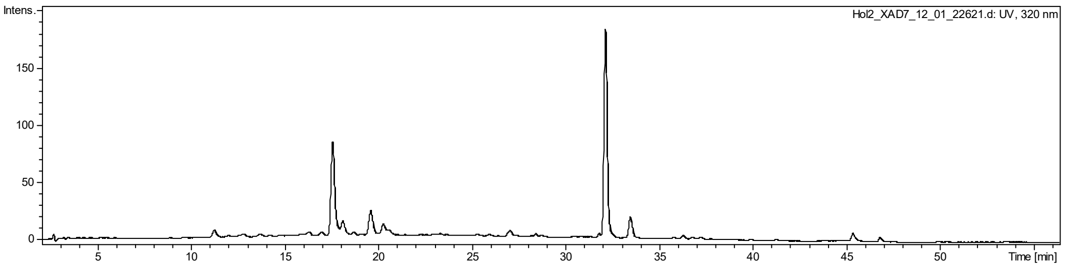

(c)

Figure S5. Isocontour plot from λ 200 to 600 (a) and DAD chromatograms at a wavelength of λ 520 (b) and λ 320 (c) of the high-performance liquid chromatography electrospray ionization mass spectrometry (HPLC-ESI-MS/MS) chromatograms of the XAD 7 extract of EB1. Peak identification is given in Tables 9 and 10.

Table S9. HPLC-ESI-MS/MS data of the XAD 7 extract of EB1, identified anthocyanins and their absorption maxima λ_{max}.

Peak No.	Retention time (min)	$[M+H]^+$ *m/z*	Fragments *m/z*	λ_{max}	Anthocyanin	Identification
1	14,9	611	287	526	Cyanidin-dihexoside	B
2	16,0	743	287	514	Cyanidin-3-sambubioside-5-glucoside	B
3	20,4	581	287	516	Cyanidin-3-sambubioside	B
4	20,8	449	287	516	Cyanidin-3-glucoside	A

Compounds were identified by (A) mass spectral data and comparison with authentic reference, (B) mass spectral data and literature data or (C) mass data only.

Table S10. HPLC-ESI-MS/MS data of the XAD 7 extract of EB1, identified copigmets and their absorption maxima λ_{max}.

Peak No.	Retention time (min)	$[M-H]^-$ *m/z*	Fragments *m/z*	λ_{max}	Copigment	Identification
1	10,9	353	191	280, 320	Neochlorogenic acid	A
2	17,1	353 (707)	191	324	Chlorogenic acid	A
3	17,9	353	191	284, 319	Cryptochlorogenic acid	A
4	19,2	625	417, 463→301	285, 319	Quercetin-dihexoside	B
5	19,6	771	609→301	-	Quercetin-derivative	C
6	32,3	609	301	265, 352	Quercetin-3-rutinoside	A
7	33,7	463	301	255, 352	Quercetin-3-glucoside	A
8	36,5	593	285	265, 325	Kaempferol-3-rutinoside	B
9	37,4	623	315	338	Isorhamnetin-rutinoside	B
10	47,0	301	151	368	Quercetin	A

Compounds were identified by (A) mass spectral data and comparison with authentic reference, (B) mass spectral data and literature data or (C) mass data only.

Red Grape

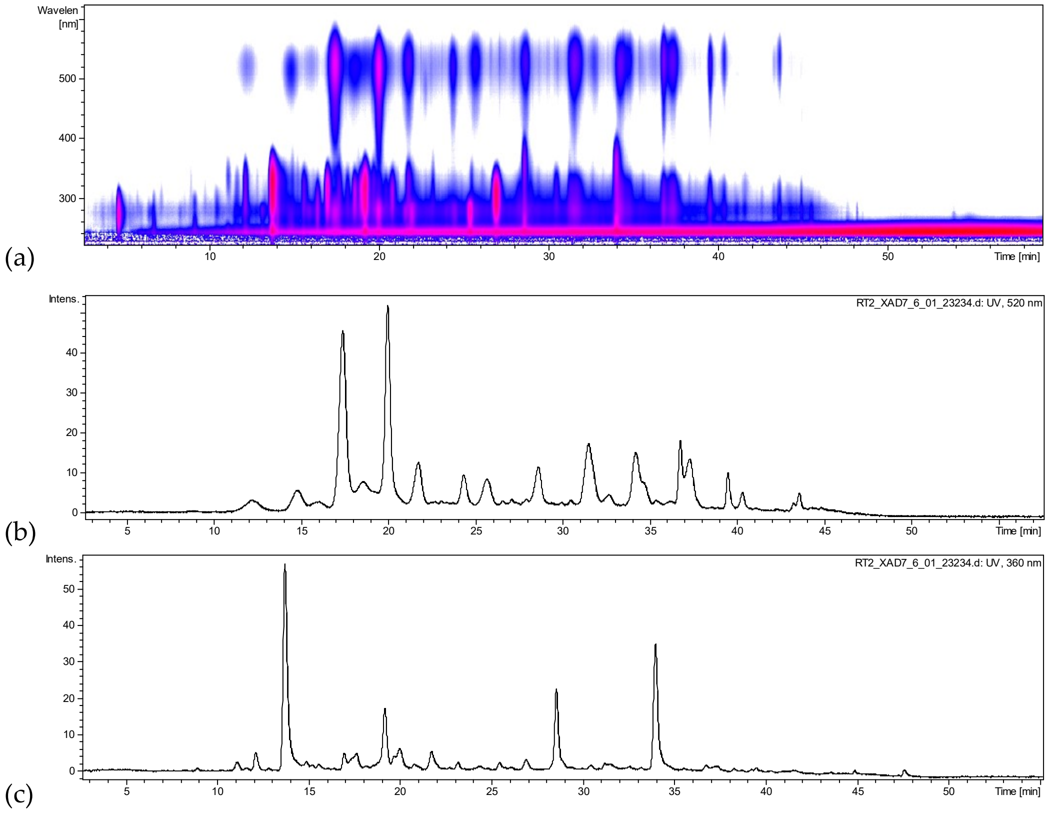

Figure S6. Isocontour plot from λ 200 to 600 (a) and DAD chromatograms at a wavelength of λ 520 (b) and λ 360 (c) of the high-performance liquid chromatography electrospray ionization mass spectrometry (HPLC-ESI-MS/MS) chromatograms of the XAD 7 extract of RG2. Peak identification is given in Tables 11 and 12.

Table S11. HPLC-ESI-MS/MS data of the XAD 7 extract of RG2, identified anthocyanins and their absorption maxima λ_{max}.

Peak No.	Retention Time (min)	[M+H]$^+$ *m/z*	Fragments *m/z*	λ_{max}	Anthocyanin	Identification
1	12,4	627	303	518	Delphinidin-dihexoside	C
2	14,9	611	287	514	Cyanidin-dihexoside	C
3	16,0	641	317	519	Petunidin-dihexoside	C
4	17,4	465	303	522	Delphinidin-3-glucoside	A
5	18,6	625	301	515	Peonidin-dihexoside	C
6	20,0	449	287	515	Cyanidin-3-glucoside	A
7	21,7	479	317	523	Petunidin-3-glucoside	A
8	24,4	463	301	517	Peonidin-3-glucoside	A
9	25,8	493	331	525	Malvidin-3-glucoside	A
10	28,7	507	303	525	Delphinidin-3-(6′′-acetoyl)glucoside	B
11	31,3	491	287	523	Cyanidin-3-(6′′-acetoyl)glucoside	B
12	32,0	773	303	527	Delphinidin-3-(6′′-coumaroyl)-5-diglucoside	B

13	32,6	521	317	523	Petunidin-3-(6´´-acetoyl)hexoside	B
14	34,2	757	595, 287	522	Cyanidin-3-(6″-coumaroyl)-5-diglucoside	B
15	34,8	787	317, 625	530	Petunidin-3-(6″-coumaroyl)-5-diglucoside	B
16	36,8	611	303	527	Delphinidin-3-(6″-coumaroyl)hexoside	B
17	37,3	801	331, 639	525	Malvidin-3-(coumaroyl)-5-diglucoside	B
18	39,6	595	287	522	Cyanidin-3-(6″-coumaroyl)glucoside	B
19	40,4	625	317	530	Petunidin-3-(6″-coumaroyl)glucoside	B
20	43,4	609	301	530	Peonidin-3-(6″-coumaroyl)hexoside	B
21	43,6	639	331	530	Malvidin-3-(6″-coumaroyl)hexoside	B

Compounds were identified by (A) mass spectral data and comparison with authentic reference, (B) mass spectral data and literature data or (C) mass data only.

Table S12. HPLC-ESI-MS/MS data of the XAD 7 extract of RG2, identified anthocyanins and their absorption maxima λ_{max}.

Peak No.	Retention Time (min)	[M-H]$^-$ *m/z*	Fragments *m/z*	λ_{max}	Copigment	Identification
1	5,1	169	125	-	Gallic acid	A
2	13,8	577	407	327	Proanthocyanidin dimer	B
3	17,1	325	145	314	Coumaric acid hexoside	B
4	19,2	295	163	313	Coutaric acid	B
5	19,8	577	407	315	Proanthocyanidin dimer	B
6	27,0	163	119	309	Coumaric acid	A
7	28,4	479	316,317	352	Isorhamnetin-hexoside	B
8	33,8	463	301	351	Quercetin-hexoside	B
9	34,1	477	301	314	Quercetin-glucuronide	B

Compounds were identified by (A) mass spectral data and comparison with authentic reference, (B) mass spectral data and literature data or (C) mass data only.

Sour Cherry

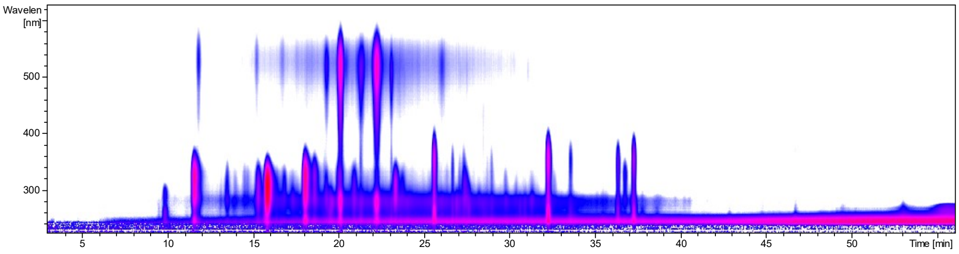

(a)

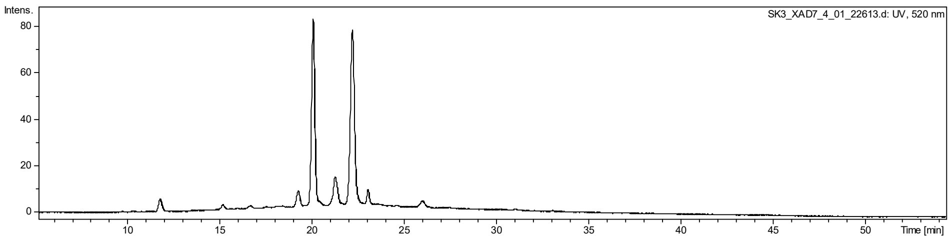

(b)

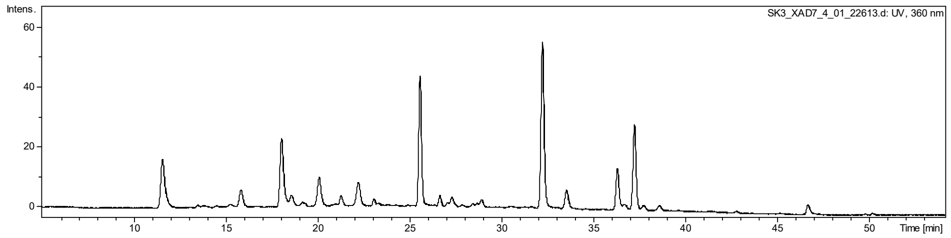

(c)

Figure S7. Isocontour plot from λ 200 to 600 (a) and DAD chromatograms at a wavelength of λ 520 (b) and λ 360 (c) of the high-performance liquid chromatography electrospray ionization mass spectrometry (HPLC-ESI-MS/MS) chromatograms of the XAD 7 extract of SC1. Peak identification is given in Table 13 and 14.

Table S13. HPLC-ESI-MS/MS data of the XAD 7 extract of SC1, identified anthocyanins and their absorption maxima λ_{max}.

Peak No.	Retention Time (min)	$[M+H]^+$ *m/z*	Fragments *m/z*	λ_{max}	Anthocyanin	Identification
1	19,3	611	287	519	Cyanidin-3-sophoroside	B
2	20,1	757	287	517	Cyanidin-3-(2^G-glucosylrutinoside)	B
3	21,6	727	287	522	Cyanidin-3-(2^G-xylosylrutinoside)	B
4	22,3	595	287	517	Cyanidin-3-rutinoside	B

Compounds were identified by (A) mass spectral data and comparison with authentic reference, (B) mass spectral data and literature data or (C) mass data only.

Table S14. HPLC-ESI-MS/MS data of the XAD 7 extract of SC1, identified copigments and their absorption maxima λ_{max}.

Peak No.	Retention Time (min)	$[M-H]^-$ *m/z*	Fragments *m/z*	λ_{max}	Copigment	Identification
1	11,6	353	191	323	Neochlorogenic acid	A
2	15,9	337	163	310	Coumaroylquinic acid	B
3	18,1	353	191	324	Chlorogenic acid	A
4	18,7	353	191	321	Cryptochlorogenic acid	A
5	20,1	577	407	320	Proanthocyanidin dimer	B
6	25,6	771	301	351	Quercetin-3-(2^G-glucosylrutinoside)	B
7	27,4	625	301	308	Quercetin-derivative	C
8	32,3	609	301	353	Quercetin-3-rutinoside	A
9	36,4	593	285	347	Kaempferol-3-rutinoside	B
10	37,3	623	315	351	Isorhamnetin-rutinoside	B

Compounds were identified by (A) mass spectral data and comparison with authentic reference, (B) mass spectral data and literature data or (C) mass data only.

Black Currant

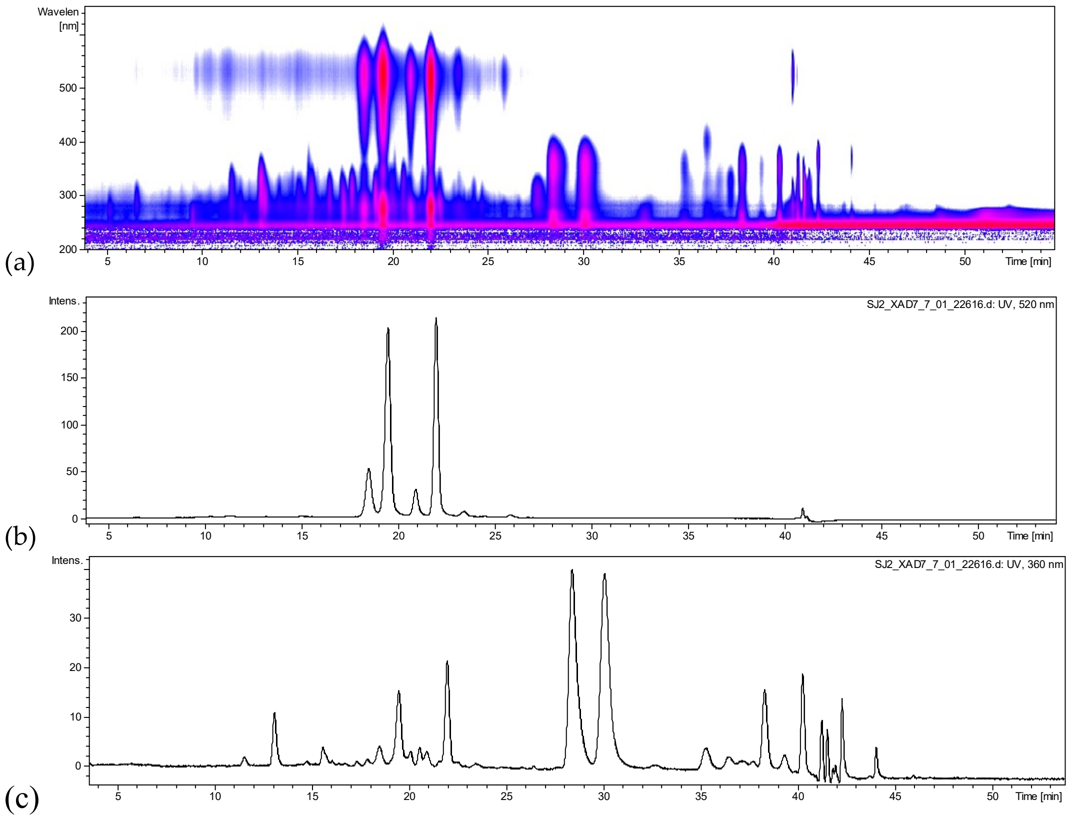

Figure S8. Isocontour plot from λ 200 to 600 (a) and DAD chromatograms at a wavelength of λ 520 (b) and λ 360 (c) of the high-performance liquid chromatography electrospray ionization mass spectrometry (HPLC-ESI-MS/MS) chromatograms of the XAD 7 extract of BC2. Peak identification is given in Tables 15 and 16.

Table S15. HPLC-ESI-MS/MS data of the XAD 7 extract of BC2, identified anthocyanins and their absorption maxima λ_{max}.

Peak No.	Retention Time (min)	$[M+H]^+$ *m/z*	Fragments *m/z*	λ_{max}	Anthocyanin	Identification
1	18,5	465	303	523	Delphinidin-3-glucoside	A
2	19,5	611	303	525	Delphinidin-3-rutinoside	B
3	20,9	449	287	515	Cyanidin-3-glucoside	A
4	22,0	595	287	516	Cyanidin-3-rutinoside	B
5	23,5	625	317	524	Petunidin-3-(6′′-coumaroyl)glucoside	B

Compounds were identified by (A) mass spectral data and comparison with authentic reference, (B) mass spectral data and literature data or (C) mass data only.

Table S16. HPLC-ESI-MS/MS data of the XAD 7 extract of BC2, identified copigments and their absorption maxima λ_{max}.

Peak No.	Retention time (min)	$[M-H]^-$ *m/z*	Fragments *m/z*	λ_{max}	Copigment	Identification
1	13,2	341	179	319	Caffeic acid hexoside	B
2	15,7	627	301,475	306	Quercetin-derivative	C
3	18,1	325	145, 163	344	Coumaric acid hexoside	B
4	19,1	463	301	297	Quercetin-hexoside	B
5	20,6	609	301	326	Quercetin-derivative	C
6	28,4	625	317	355	Isorhamnetin-rutinoside	B
7	30,0	625	317,179	355	Isorhamnetin-derivative	C
8	38,4	609	301	352	Quercetin-3-rutinoside	A
9	40,1	463	301	352	Quercetin-3-hexoside	B
10	41,2	609	301	349	Quercetin-derivative	C
11	41,4	593	285	318	Kaempferol-rutinoside	B

Compounds were identified by (A) mass spectral data and comparison with authentic reference, (B) mass spectral data and literature data or (C) mass data only.

© 2020 by the authors. Licensee MDPI, Basel, Switzerland. This article is an open access article distributed under the terms and conditions of the Creative Commons Attribution (CC BY) license (http://creativecommons.org/licenses/by/4.0/).
